# Robotic Resection of a Papillary Fibroelastoma Arising From the Coumadin Ridge: A Multimodality Imaging of an Echocardiographic Blind Spot

**DOI:** 10.7759/cureus.104382

**Published:** 2026-02-27

**Authors:** Daiki Yoshiyama, Norihiko Ishikawa, Erisa Kojima, Kazuto Miyata, Go Watanabe

**Affiliations:** 1 Cardiovascular Surgery, NewHeart Watanabe Institute, Tokyo, JPN; 2 Clinical Laboratory, NewHeart Watanabe Institute, Tokyo, JPN; 3 Anesthesiology, NewHeart Watanabe Institute, Tokyo, JPN

**Keywords:** coumadin ridge, echocardiography, multimodality imaging, papillary fibroelastoma, robotic cardiac surgery

## Abstract

Papillary fibroelastomas (PFEs) are rare, benign cardiac tumors that carry a significant risk of cardioembolism. While transthoracic echocardiography (TTE) is the standard initial screening tool, certain anatomical locations, such as the coumadin ridge, can present diagnostic challenges. We report the case of a PFE located in an echocardiographic "blind spot" that required a multimodality imaging approach for diagnosis and surgical planning. A 61-year-old woman was referred to our hospital after a 5-mm mass near the left atrial appendage was incidentally detected on cardiac computed tomography angiography (CCTA). The patient had no history of embolic events. Notably, standard TTE failed to visualize the mass. A comprehensive multimodality assessment was performed: transesophageal echocardiography (TEE) revealed a highly mobile mass attached to the coumadin ridge with moderate mitral regurgitation; CCTA clarified the anatomical relationship; and cardiac magnetic resonance imaging findings were more consistent with a tumor than a thrombus. Given the high embolic risk posed by the tumor's mobility, a robotic minimally invasive resection and mitral repair was performed. The tumor was successfully excised without complications, and histopathology confirmed a PFE. This case highlights the limitations of TTE in evaluating the coumadin ridge and underscores the indispensability of multimodality imaging for characterizing small, mobile cardiac tumors located in anatomical blind spots. Robotic surgery offers a safe and effective minimally invasive treatment option for preventing embolic complications in such cases.

## Introduction

The coumadin ridge (or left atrial ridge) is a prominent muscular fold separating the left atrial appendage from the left superior pulmonary vein. While it is a normal anatomical structure, it can occasionally be the site of cardiac masses, creating a diagnostic dilemma between thrombus, vegetation, and tumors [1]. Among primary cardiac tumors, papillary fibroelastoma (PFE) is the second most common benign neoplasm after myxoma, yet it carries a disproportionately high risk of life-threatening cardioembolic complications such as stroke, myocardial infarction, and peripheral embolization [2-4].

A thrombus located in the postero-lateral region of the left atrium is hard to differentiate from the left atrial ridge. Historically, this phenomenon has been associated with considerable abuse of anticoagulants in patients with an incidentally detected left atrial ridge. Nevertheless, the prominent ridge may coexist with a tightly adhering thrombus; thus, multimodal imaging should be performed to exclude any emboli located in this region of the left atrium [5]. Furthermore, standard transthoracic echocardiography (TTE) has limitations in visualizing small lesions in this area due to anatomical shadowing, leading to potential misdiagnoses or missed opportunities for early intervention [1,6]. Once a mass is identified, differentiating a PFE from a thrombus is also of paramount importance, as their management strategies diverge significantly: anticoagulation for thrombi versus surgical resection for tumors.

While surgical resection is the established standard of care for symptomatic PFEs, management of small, asymptomatic PFEs is debated; some advocate surveillance in selected low-risk cases, whereas others favor resection when lesions are left-sided and/or highly mobile [3,7]. Historically, a conservative approach with anticoagulation was favored for asymptomatic patients to avoid the morbidity associated with a traditional median sternotomy. Surgical resection of cardiac masses conventionally required this invasive approach. However, over the past two decades, robot-assisted minimally invasive cardiac surgery has emerged as a superior alternative. The robotic approach provides enhanced 3D visualization, precise articulation in confined spaces, and significantly reduced surgical trauma compared to traditional sternotomy [8]. With the advent of this technology, the risk-benefit ratio has shifted towards early, proactive resection [9].

We present the case of a highly mobile PFE on the coumadin ridge that was initially missed by TTE but successfully identified through a multimodality imaging approach. The tumor was safely resected using a robot-assisted system, highlighting the role of advanced imaging and minimally invasive techniques in managing high-risk anatomical "blind spots."

## Case presentation

A 61-year-old woman was referred to our institution for the evaluation of a 5-mm mass near the left atrial appendage, incidentally found on cardiac computed tomography angiography (CCTA) performed at the referring hospital. She had been experiencing intermittent palpitations and atypical chest pain for three months. Physical examination was unremarkable, with no murmurs or neurological deficits. The CCTA, utilizing a prospective electrocardiogram (ECG)-gated protocol, successfully ruled out significant coronary stenosis. At the time of presentation, she was not on any anticoagulation therapy and had no known risk factors for stroke. Her ECG demonstrated a normal sinus rhythm, and routine laboratory findings were unremarkable.

**Figure 1:**
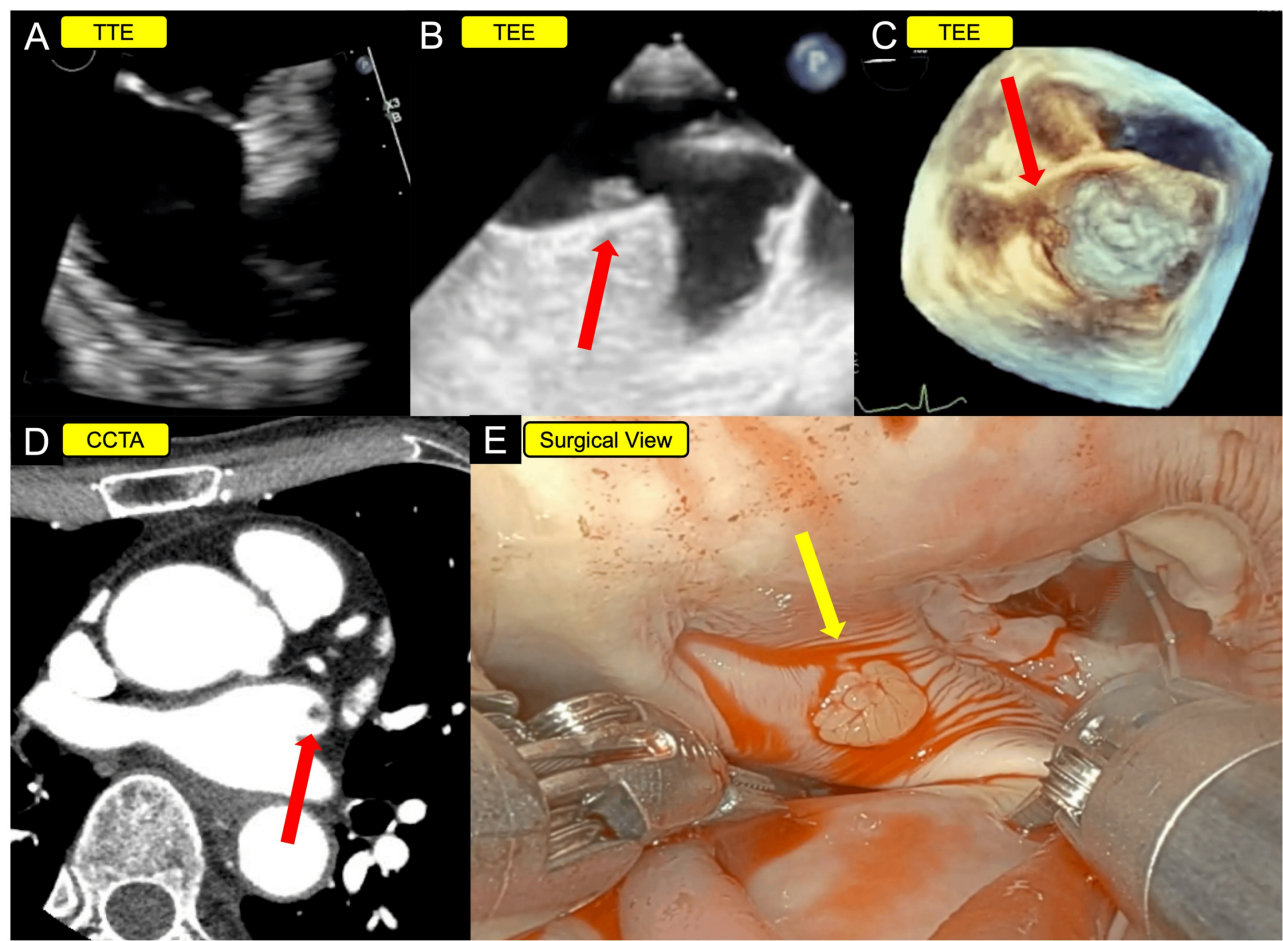
Multimodality imaging of the case (A) TTE did not reveal any obvious mass. (B, C) TEE revealed a highly mobile 5-mm mass attached to the coumadin ridge (red arrow). (D) CCTA identified a 5-mm mass near the base of the left atrial appendage (red arrow). (E) Intraoperatively, a papillary fibroelastoma was identified on the coumadin ridge (yellow arrow). TTE: transthoracic echocardiography; TEE: transesophageal echocardiography; CCTA: cardiac computed tomography angiography

Upon admission, a TTE was performed but failed to visualize the mass. Subsequent transesophageal echocardiography (TEE) successfully identified a highly mobile, 5-mm, pedunculated mass attached to the coumadin ridge, along with moderate mitral regurgitation (Figure 1, Video 1).

**Video 1:** TTE and TEE of the case TTE: transthoracic echocardiography; TEE: transesophageal echocardiography

**Figure 2:**
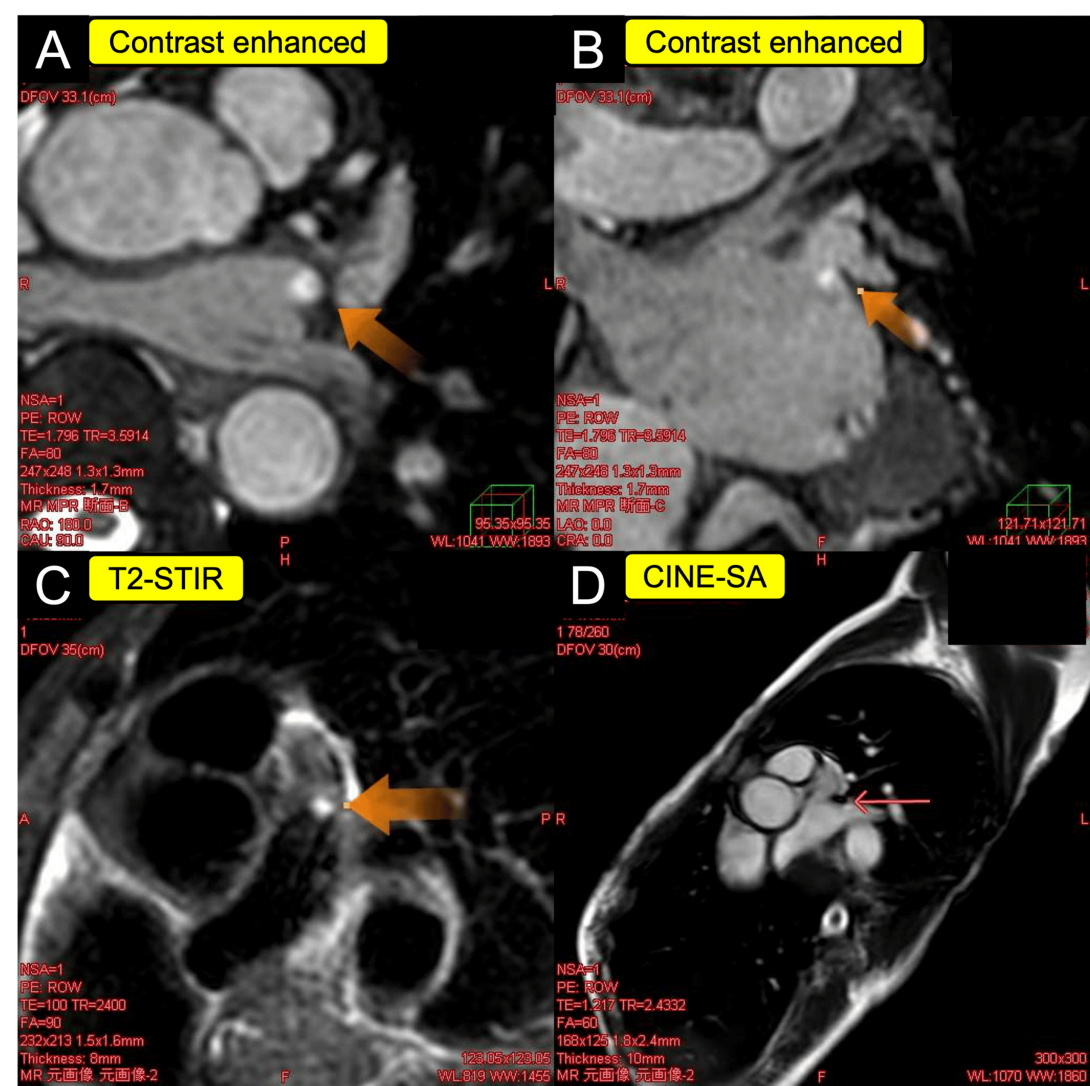
Cardiac magnetic resonance imaging of the case (A, B) Contrast enhanced showed the magnetic resonance imaging enhancement of the mass (orange arrow). (C) T2-STIR showed the hyperintense mass (orange arrow). (D) Cine SA showed the isointense mass (arrow). T2-STIR: T2-weighted short tau inversion recovery; SA: short-axis

To further characterize the lesion, cardiac magnetic resonance imaging (CMR) was performed. Given the small size of the 5-mm mass, CMR characterization was technically challenging; nevertheless, it yielded supportive findings. On cine short-axis (SA) imaging, the mass appeared isointense relative to the myocardium. Such isointense signals on cine imaging usually indicate a tissue similar in density to the heart muscle, consistent with several potential cardiac tumors. Furthermore, the mass appeared isointense on T1-weighted imaging and hyperintense on T2-weighted short tau inversion recovery (T2-STIR) imaging. T2-STIR is a specialized magnetic resonance imaging (MRI) sequence that suppresses the signal from fat while highlighting water content, making it highly effective in cardiovascular imaging for detecting tissue edema and inflammation. Following gadolinium administration, mild contrast enhancement was observed (Figure 2).

Although late gadolinium enhancement was not distinct, these features were more suggestive of a tumor, such as a PFE, than a thrombus, although a myxoma could not be entirely excluded.

Given the tumor's high mobility and inherent embolic risk, a consensus was reached to proceed with surgical resection for primary prevention. A robotic-assisted cardiac tumor resection was performed using the da Vinci X surgical system (Intuitive Surgical Inc., Sunnyvale, CA, USA). The robotic ports were placed in the right second, third, and fourth intercostal spaces, with skin incisions of 1.5 cm, 2.5 cm, and 2 cm, respectively. Cardiopulmonary bypass was established via the peripheral cannulation of the right femoral artery and vein. A left atriotomy revealed a frond-like, mobile mass attached to the coumadin ridge by a remarkably fragile stalk. The mass was easily removed with gentle traction. We trimmed the endocardium at the attachment site to prevent recurrence and subsequently performed mitral valve repair using a 28-mm semi-rigid open ring (PhysioFlex, annuloplasty ring, Edwards Lifesciences, Irvine, CA, USA). The operation was completed in 103 minutes, with a cardiopulmonary bypass time of 62 minutes, an aortic cross-clamp time of 34 minutes, and a console time of 58 minutes (Video 2).

**Video 2:** Intraoperative video of the case A papillary fibroelastoma was identified on the coumadin ridge.

Histopathological examination confirmed the diagnosis of PFE. The official pathology report described characteristic microscopic findings, including avascular papillary fronds consisting of a central core of dense elastic tissue covered by hyperplastic endothelial cells, which are pathognomonic for this tumor. The patient's postoperative course was uneventful, and she was discharged on postoperative day 7. At her six-month follow-up, to reliably exclude recurrence given the anatomical blind spot on TTE, both routine TTE and CCTA were performed, which revealed no evidence of tumor recurrence.

## Discussion

This case highlights two crucial, evolving concepts in the management of small cardiac tumors: the diagnostic power of multimodality imaging and the rationale for a lower threshold for surgical intervention.

The diagnostic imperative: beyond echocardiography for small tumors

The initial failure of TTE to detect the mass is a key teaching point. The posterior location of the coumadin ridge makes it a notorious blind spot for TTE due to far-field artifacts and acoustic shadowing [10,11]. Furthermore, this prominent anatomical structure is frequently mistaken for a thrombus. Historically, such misdiagnoses resulted in unnecessary anticoagulation with warfarin, which is precisely how the structure earned the name "coumadin ridge" [12]. However, with the evolution of cardiac imaging and surgical techniques, this conventional approach warrants re-evaluation. This case, in which TTE failed to identify the lesion despite prior suspicion on CCTA, supports the understanding that a negative TTE cannot reliably exclude pathology or may underestimate the lesion in this specific region. Furthermore, for small masses (<10 mm), a critical differential diagnosis is between a PFE and a thrombus [3]. While some older literature might suggest a trial of anticoagulation, this approach is fraught with risk, as even sub-centimeter PFEs have been reported to cause devastating embolic events [2]. Therefore, establishing a more definitive diagnosis is crucial. Our case demonstrates the synergistic value of an integrated imaging approach: CCTA provided high-spatial-resolution anatomical delineation, TEE assessed tumor mobility and attachment, and CMR was instrumental in tissue characterization. The CCTA enhanced diagnostic accuracy and provided the precise anatomical localization necessary for surgical planning [12,13]. Given the small 5-mm size of the lesion, CMR findings were supportive of a neoplastic lesion rather than a thrombus [13,14]. In anatomically challenging locations, relying on a single modality may be insufficient; instead, a multimodal imaging strategy that leverages the complementary strengths of TEE, CCTA, and CMR is pivotal to overcome the limitations of TTE and establish a definitive diagnosis [15,16].

Re-evaluating surgical indications in the era of minimally invasive surgery

The management of asymptomatic PFEs for primary prevention remains a subject of debate. While previous reports traditionally considered a size of 10 mm or larger as the threshold for surgery based on historical operative risks [16,17], emerging evidence suggests that intervention should be considered regardless of size [17,18]. Our intraoperative finding of a 5-mm tumor attached by a fragile, thin stalk underscores that embolic potential is determined primarily by tumor morphology, specifically mobility and stalk fragility, rather than diameter alone. This fragility implies that even small tumors carry a high risk of embolization. Consequently, considering the evolution of surgical techniques, particularly robotic-assisted surgery which has minimized invasiveness and reduced morbidity [8,9], we advocate for adopting a proactive strategy of early resection. The proven safety and efficacy of these modern techniques may support a proactive surgical approach for primary prevention to mitigate the unpredictable and potentially catastrophic risk of embolism, even in small, asymptomatic cases.

## Conclusions

This case of a robotically resected PFE on the coumadin ridge illustrates the limitations of relying on a single imaging modality or traditional size-based management criteria. A definitive diagnosis of small cardiac masses in challenging locations requires a comprehensive multimodality imaging approach to accurately differentiate neoplasms from thrombi. Specifically, when evaluating lesions on the coumadin ridge, TEE is highly valuable, as this structure is best seen using the midesophageal two-chamber view. Furthermore, given the reduction in cardiac surgical risks and the minimally invasive nature of procedures such as robotic surgery, earlier surgical intervention might be considered as a primary prevention strategy, taking into account tumor mobility in addition to size. However, as this report is based on a single case, further large-scale studies are warranted to establish definitive management guidelines for small, asymptomatic lesions.
